# Report of the Committee on Surgery, to the Illinois State Medical Society

**Published:** 1863-08

**Authors:** E. Andrews

**Affiliations:** Chicago


ARTICLE XX.

### REPORT OF THE COMMITTEE ON SURGERY, TO THE ILLINOIS STATE MEDICAL SOCIETY.

BY PROFESSOR E. ANDREWS, of Chicago.

The department of surgery has made some important advances since our last meeting, which your Committee deem it important to note and discuss. Not only have we the ordinary improvements of peace to chronicle, but also the bloody experience of war. It will, therefore, be advisable to divide this Report between the two topics of Civil and Military Surgery.

### CIVIL SURGERY.

To a State Medical Society it is appropriate to recount, first, such improvements as have originated within our own borders. Among these, we have observed a new mode of treating ununited fractures, devised by Dr. PRINCE, of Jacksonville. The principle is applicable only to oblique fractures; and consists in a mode of making pressure of the two fragments against each other laterally, without compressing the soft parts. For this purpose, a strong steel semi-circle is constructed of a size rather more than sufficient to embrace the limb to which it is to be applied. From either end a narrow conical point projects towards the centre, far enough to reach the bone. One of these advances and recedes by means of a thumb-screw. In the use of the instrument, a puncture is made on opposite sides of the limb, in such a position that one puncture shall come down upon one fragment of broken bone and the other upon the other, at the place where they lap by each other. Into these incisions the two points of steel are inserted, and the extremities carefully placed upon the bone. The movable point is then screwed firmly in, by which means the fragment of bone against which it rests is pressed against its fellow with great force, and held there with entire security. In this way, excellent cures have been made with very little inconvenience to the patient or surgeon.

Some improvements have been proposed in the treatment of ordinary fractures. Professor Andrews, of Chicago, has devised an instrument for making counter-extension by adhesive-straps. It consists of an iron rod, four feet long, which is cut to a screw the whole length. This slides into a brass tube of the same length, the distance to which it goes being determined by turning a nut upon the screw. The upper end of the tube is continued by a steel bow, which is intended to curve in front of the shoulder, and bears a hook at the extremity, which lies just above the top of the shoulder. A cross-bar at the foot of the instrument is attached to the leg in the ordinary way by adhesive straps for extension. The counter-extension is then made as follows:—Three large adhesive-straps are cut, two of which are two yards each in length and three inches in width. A third is provided, a yard and a-half in length, and of the same width as the former. One of the long straps is applied from the front of the abdomen, on the same side as the fracture, straight up to the shoulder; it is there turned straight down the back, leaving two inches of slack at the top of the shoulder. The other long strap is applied as follows:—One end is placed obliquely across the trochanter of the sound side, the strap is then carried obliquely up and across the abdomen and chest, meeting the other strap at the shoulder; it is there turned down the back obliquely, leaving a slack as before, and terminates by returning across the trochanter at the starting-point. The third strap is then put as a belt around the waist to keep the others in place. The hook, at the top of the instrument, is then made to seize the slack of the straps above the shoulder, and the cross-bar at the bottom is attached to the straps at the foot. Then, by turning the nut on the screw, extension is made to any amount desired. The counter-extension thus applied, is distributed over so large a surface that it is absolutely painless, and does away entirely with the suffering occasioned by the perineal band. Dr. DODGE, of Janesville, has improved this instrument, by adding a foot-piece, and also by making the bow of spring-steel, so as to have the advantage of its elasticity.

The treatment of diseases of the knee and hip-joints has made immense advances within three years. Dr. H. G. DAVIS, of New York, has been more active in this department than any other man. Without going into the history of the matter, suffice it to say, that the following is the best system of treatment, in view of all the latest discoveries:—Both these joints are managed in the same manner. If the disease is seen in the first stages, a splint is applied at once, which is contrived to make counter-extension by an elastic perineal band, and extension by adhesive-straps, the splint itself being accurately applied to the outer side of the limb, and the lower extremity of it strapped close against the leg. A suitable extension is then made by an endless screw, which being turned, the joint is drawn out, so that the inflamed surfaces of the bones no longer press or rub on each other. In this way, the chief cause of the continuance of the disease is removed, and most of the cases recover. The patient is, at the same time, put upon tonics. Professor ANDREWS, of Chicago, has improved the splint, by having it pass up upon the inner side of the limb, and making the counter-extension not by a perineal band, which is apt to hurt, but by a broad padded crutch piece accurately moulded to fit the perineum and nates. The lower extremity passes quite down to the ground, where it is attached to the sole of the shoe. Adhesive-strap extension is used as before; and, in walking, the weight of the body is not brought upon the limb, but transmitted directly through the instrument to the ground. There is also this additional advantage, that, in case of hip-disease, if it proceeds to the second stage, the splint being upon the inner side of the limb, is not soiled by the discharges of the ulcers nor in the way of dressing them.

The treatment of erysipelas has, during the past winter, received some special attention. Dr. FISHER, of Chicago, has made use of the sulphites of lime and soda with striking success. His experiments were prompted by the investigations of Dr.

POLLI, of Milan, Italy. Dr. POLLI operated upon nearly seventy dogs, producing pyæmia artificially, by injecting putrid pus and blood into their viens, and treating them with the sulphites. These tests were prompted by the well-known power of the sulphites in arresting fermentation, combined with the belief that erysipelas, pyæmia, hospital gangrene, etc., were zymotic or fermentive in their pathology. The results of Dr. POLLI'S operations, if correctly reported, show a surprising power in the sulphites over this class of complaints, but he does not appear to have ventured his treatment upon the human subject. Dr. FISHER administers the article in drachm doses, repeated every three or four hours. He prefers the sulphite of soda when the bowels are constipated, and the sulphite of lime when they are too lax His belief is, that the disease is perceptibly mitigated in twenty-four hours; and, in all cases thus far, a rapid recovery has ensued. Professor DAVIS, of Chicago, has repeated the experiments with favorable results.

Many improvements have been proposed in ophthalmic surgery. Among these, the frequent use of iridectomy, or cutting out portions of the iris, in cases of glaucoma, has attracted most attention. The theory is, that glaucoma is caused by excessive secretion of the fluids within the eye, by which it is rendered too tense, and its contents are injuriously pressed upon. An incision is made near the edge of the cornea, and a portion of the iris, amounting to about one-third, is drawn out through it and snipped off with the scissors. As the iris is the most active secreting organ in the eye, the production of fluid is thus abated, the pressure diminished, and the evil cured or mitigated. There is no doubt of the frequent benefits derived from this operation, but the mania for its performance, which manifests itself among some writers, needs a little check, lest it lead to abuses.

Spinal diseases are much more successfully managed than formerly, especially curvatures. The apparatuses now used are much more efficient than those formerly constructed; and we cure many patients whom we formerly considered as doomed to helpless and hopeless deformity.

MILITARY SURGERY.

Illinois has contributed her medical men liberally to the necessities of the war. These men all passed a rigid examination before a Board appointed for the purpose, and a large number of incompetent men were thus prevented from obtaining positions. In consequence of the faithfulness of the examiners, the medical officers of the Illinois troops stand very high in respect to skill and capacity.

Under the first call for troops, the Board of Examiners consisted of

Professor N. S. DAVIS, of Chicago,
Dr. C. RYAN, of Sangamon Co.,
Dr. G. W. STIPP, of McLean Co.,
Dr. WM. CHAMBERS, of Coles Co.,
Dr. CARPENTER, of Coles Co.

At the second call, a new Board was appointed, which, by occasionally filling vacancies, has been continued in full force to the present time. The names of the gentlemen who have been or are now members of it are as follows:—

Professor H. A. JOHNSON, of Chicago,
Dr. H. W. DAVIS, of Paris,
Professor H. WING, of Chicago,
Dr. O. M. BRYAN, of Sycamore,
Dr. R. ROSKOTTEN, of Peoria,
Professor D. BRAINARD, of Chicago,
Dr. D. K. GREEN, of Salem,
Professor A. L. MCARTHUR, of Joliet.

Up to January 1st, the Board had examined 595 candidates. Of these, 259 were recommended for Surgeons, 266 for Assistant-Surgeons, and 70 were rejected.

We are gratified to state, that the surgeons from this State have, generally, done themselves honor.

As a portion of your Committee has been engaged in active field service during the year, we are enabled to present to the Society some conclusions which we have derived from a large number of gun-shot wounds, with the operations and results consequent upon them. Much of this information we obtained by personal observation upon the field, and all of it from thoroughly reliable sources.

The wounds were distributed through the body in the following proportions:—

| | | |
|---|---|---|
| Wounds of the Head, | | 50 |
| do. | Neck, | 10 |
| do. | Trunk, (not including pelvis,) | 164 |
| do. | Arm, | 69 |
| do. | Elbow, | 14 |
| do. | Fore-arm, | 43 |
| do. | Hand, | 77 |
| do. | Hip, | 43 |
| do. | Thigh, | 109 |
| do. | Knee, | 26 |
| do. | Leg, | 79 |
| do. | Foot, | 50 |
| Total, | | 734 |

*Injuries of the Head.*—We saw great numbers of these in different battles, of whom we could obtain no record. Our recorded cases are 50 in number, which were distributed as follows: flesh wounds and contusions 30, fractures of the face 9, fractures of the cranium 5. The small number of fractures of the cranium results from the following causes: 1st, many wounded in the brain die on the spot, and never appear before the surgeon; 2d, the face lying in front of the cranium, often shields it; 3d, many bullets, striking the cranium obliquely, glance off, merely plowing the scalp. Of these 5 fractures, 2 were from bullets penetrating the brain, and 3 from pieces of shell or oblique bullets. They all died without exception; only 1 was trepanned, and he without benefit. The general result in military surgery is, that gun-shot fractures of the cranium are fatal, and that trepanning is very seldom useful. In penetrating wounds of the brain, the bullet drives before it numerous fragments of bone, hair, clothing, etc., which lodge in the cerebral substance, and occasion hopeless inflammation. A few unrecorded cases of recovery, however, came to our knowledge, and it is worthy of notice that these were, without exception, wounds of the anterior lobe of the brain, which, for some reason seems to sustain injury with less mortality than any other part.

Of the 9 fractures of the face 5 recovered, 1 died, and 3 remained in a doubtful state. Bullet wounds in the bones of the face are somewhat prone to be followed by secondary hæmorrhage.

Of the 30 flesh wounds, 16 recovered, 4 died, and 10 remained doubtful. Of the entire 50 wounds of the head, of all kinds, 26 recovered, 10 died, and 14 remained uncertain.

*Wounds of the Neck.*—These were 10 in number, and were all flesh wounds; 6 recovered, and 4 remained in doubt. Wounds of the large vessels, and fractures of the cervical vertebræ usually die on the field, at once, without coming to the notice of the surgeon.

*Wounds of the Trunk.*—Under this head we include the shoulder, but reserve the hips for a separate consideration, as thus considered, the wounds of the trunk were 164 in number; 36 penetrated the lungs, 10 pierced the cavity of the abdomen, 31 were flesh or fracture wounds of the shoulder, and 87 were flesh wounds of various regions, or fractures of ribs, not penetrating any cavity.

Of the 36 wounds of the lung, 12 recovered, 18 died, and 6 were uncertain.

Of the 10 wounds penetrating the cavity of the abdomen, 2 were stabs, and 8 gun-shot wounds. The stabbed cases both recovered; but of the 8 bullet wounds, 6 died, and 2 remained in doubt. There was very little hope of them, however, and they should, probably, all be reckoned as dead. With very few exceptions, bullet wounds into the abdominal cavity are all fatal. It may be a question worthy of serious thought, in view of the hopelessness of our present practice, whether we ought not to cut boldly into the abdominal cavity, wash out the filth, and, bringing the wounded intestine to the surface, endeavor to produce an artificial anus.

Of the wounds of the shoulder, 31 in number, 20 recovered, 2 died, and 11 remained in doubt.

The 87 superficial wounds of the trunk all recovered.

Of the total number of those wounded in the trunk and shoulder, 20 died, 142 recovered, and 2 were doubtful.

Wounds of the head, neck, and trunk, from their nature, seldom admit of much surgical assistance; taken as one class, they present a mortality of about 20 or 30 per cent; which may be somewhat diminished by good care, or horribly increased by bad air in a crowded hospital; but can be little affected by operative measures, except in a few instances.

*Wounds of the Arm.*—The very opposite is true, however, of the wounds of the extremities; here the skill and sound judgment of the operator are of immense value, and the correctness or error of his measures will produce vast changes in the ratio between mortality and recovery.

Of wounds of the arm, our records show 69 cases, of which 28 were compound fractures of the humerus, and 41 were flesh wounds. The flesh wounds all recovered; of the fractures, 21 recovered, 4 died, and 3 were in doubt. In 6 of the fractured cases, the shoulder-joint was resected; of which, 5 recovered, and 1 died. In 6 others, amputation was performed at the shoulder-joint; of which, 4 recovered, and 2 died. In 8 cases, amputation of the arm was performed; of which, 7 recovered, and 1 is unknown. In 8 cases, no operation was performed, and the fracture was treated with splints; of these, 7 recovered and 1 died.

The ratio of mortality in all the gun-shot fractures of the humerus is 1 in 7. The question of the grounds of choice, between resections and amputations of the extremities, will be discussed below, under the head of operations.

*Wounds of the Elbow.*—Of these, 4 were flesh wounds, of which, 2 recovered, and 2 are unknown; 10 cases were compound fractures of the joint, of which, 7 recovered, 1 died, and 2 remained undecided. In 4 of the cases, resection of the joint was performed, of which, 3 recovered, and 1 died. In 3 cases, amputation of the arm was resorted to, of which, 2 recovered, and 1 was not decided. In 3 cases of less severity, no operation was performed, and all recovered.

The total number of wounded in the elbow was 14; of whom, 9 recovered, 1 died, and 4 remained doubtful.

*Wounds of the Fore-arm.*—Of these, 27 were flesh wounds, and 16 were compound fractures. Of the flesh wounds, 22 recovered, and 5 were doubtful. Of the compound fractures, 10 recovered, and 6 remained in doubt.

In 4 of the cases, amputation was performed, and all of them recovered; no death, therefore, was observed from wounds of the fore-arm.

*Wounds of the Hand.*—Of these, 38 were flesh wounds, of which, 37 recovered, and 1 died; 25 cases were fractures of the phalanges, of which, 18 recovered, and 7 are unknown; 9 cases were fractures of the metacarpals, of which, 4 recovered, and 5 are unknown; 5 cases were fractures of the wrist, of which, 3 recovered, and 2 are doubtful. 24 fingers were amputated, of which cases, 19 recovered, and 5 were not heard from. One amputation was performed through the metacarpals,—result unknown. One shot across the metacarpals was very unjustifiably treated by amputation of the fore-arm, four inches above the injury; the patient recovered.

Total wounds of the hand, 77; known mortality, 1.

*Wounds of the Pelvic Region.*—40 flesh wounds of this region occurred, of which, 30 recovered, 3 died, and 7 were undecided; 1 of the 3 cases which died was wounded in the bladder, another perished of secondary hæmorrhage and general exhaustion, from the bad air of an overcrowded boat, and the third died suddenly from cause unknown.

Only 3 cases of fracture of the pelvis were brought to our notice, 2 of which recovered, and 1 died; the viscera were not wounded in either. Total wounds of the pelvic region, 43.

*Wounds of the Thigh.*—This is a most important division of the field of military surgery, and from it spring some of the most trying and difficult questions which are ever laid before the operator for decision. The discussion of these questions will be given below, under the head of operations.

The total number of wounds of the thigh was 109, of which, 90 were flesh wounds, and 19 were compound fractures. Of the

90 flesh wounds, 76 recovered, 3 died, and 11 were doubtful; of the 19 fractures, 6 recovered, 12 died, and 1 was doubtful; 5 of the fractured cases were amputated at the upper third, of which, 1 recovered, and 4 died; 3 were amputated at the middle third, of which, 2 recovered, and 1 died; 1 was amputated at the lower third, and recovered; 2 cases were treated by resecting the fractured portions in the continuity of the shaft, both of these died; 9 cases were treated without operative interference, by simply employing splints, position, and such incisions as were necessary to evacuate pus, of these, 3 recovered, and 6 died. 2 of those which recovered were shot in the cancellar tissue of the neck or trochanter, where any operation must necessarily have been amputation at the hip, or excision of the head of the bone; 1 of them lay twenty hours on the field, in very raw and cold weather. It would seem that shots through the cancellar tissue, at the superior fifth of the femur, are much less dangerous than those in the compact bone of the shaft below; the reason is, that when a ball bores its way through spongy bone, it produces only a moderate amount of shattering, owing to the yielding character of that tissue; but the impact of a minnie bullet upon the brittle ivory of the shaft, shatters it for several inches, and disperses the fragments with the force of an explosion among all the surrounding tissues, producing immense disorganization. These cases nearly all die within the first five days, no matter what treatment is adopted.

*Wounds of the Knee.*—There were 26 wounds of the region of the knee, of these, 14 were flesh wounds, and 12 were compound fractures; 12 of the flesh wounds recovered, none died, and 2 remained doubtful. Of the 12 compound fractures, 5 recovered, 4 died, and 3 remained doubtful; 10 of these fractures were treated by amputation at the lower third of the thigh, of which, 6 recovered, 3 died, and 1 remained in doubt; 1 case was treated by resection of the knee-joint, and recovered; 1 was treated without any operation, and died. In this connection, it may be remarked, that we observed a considerable number of cases of gun-shot fractures of the knee at the battle of Shiloh, very injudiciously treated as ordinary fractures, without any operation; as we could obtain no record of the cases, we have not entered them in the lists, but we never knew one to recover. Let any young surgeon, who is reluctant to sacrifice the limb or joint in these cases, take the trouble to dissect two or three of them, and he will see at once why they all die, unless they are amputated or resected. The bullet disorganizes the interior of the joint in a most surprising manner, filling it with five hundred fragments of bone and cartilage and putting it in a condition from which no human frame can recover without operative help.

*Wounds of the Leg.*—These were 79 in number, of which, 56 were flesh wounds, and 23 were fractures. Of the 56 flesh wounds, 51 recovered, 1 died, and 4 were undecided; of the 23 cases of fracture, 14 recovered, 7 died, and 2 are unknown; 12 of the fractures were treated by amputation of the leg, of which, 11 recovered, and 1 died; 1 was treated by amputation of the lower third of the thigh, and recovered; in 1 case, a portion of the bone was resected, which also recovered; 8 cases were treated by splints, without any operation, of these, 2 recovered, 4 died, and 2 remained doubtful.

*Wounds of the Foot.*—These were 50 in number; 31 were flesh wounds, and all recovered; 4 were fractures of the phalanges, and all recovered; 6 were fractures of the metatarsus, of which cases, 4 recovered, 1 died, and 1 is unknown; 9 were fractures of the tarsus, of which, 7 recovered, 1 died, and 1 remained doubtful; amputation of the toes was performed in 4 cases, which all recovered. No amputation through the metatarsus occurred; one amputation through the tarsus was performed, and the patient recovered. In 4 cases the leg was amputated, of which, 3 recovered, and 1 died. A portion of the tarsus was resected in 1 case, which recovered.

*Predominance of wounds on the Right Side of the Body.*—In western warfare, the constant occurrence of battles in the forest gives predominance to the operations of skirmishers, who deliver their fire usually from the right hand side of the trees that shelter them; in consequence of this, the right hand, arm, and shoulder, and the right thigh, knee, and leg, receive many more wounds than the left.

*Discussion of the Operations.*—The operations in these cases were, for the most part, executed by well educated and skilful men, so that there was little occasion to criticise them. In respect to the mode of their performance, they will compare favorably with similar operations in any other army. There were some errors of judgment, respecting the kinds of treatment to be decided upon, but not more than was to be expected.

The following tables show the number and locality of the operations:—

*Amputations.*

| | Recover'd. | Died. | Doubtful. | Total. |
|---|---|---|---|---|
| Amputations at the shoulder-joint, | 4 | 2 | | 6 |
| do. of the arm, | 9 | | 2 | 11 |
| do. do. fore-arm, | 5 | | | 5 |
| do. do. hand, | 1 | | | 1 |
| do. do. fingers, | 19 | | 5 | 24 |
| do. do. thigh, upper third, | 1 | 4 | | 5 |
| do. do. do. middle do. | 2 | 2 | | 4 |
| do. do. do. lower do. | 7 | 3 | 1 | 11 |
| do. do. leg, | 14 | 2 | | 16 |
| do. do. foot, | 1 | | | 1 |
| do. do. toes, | 4 | | | 4 |
| Total, | 67 | 13 | 8 | 88 |

No case occurred in which we felt justified in amputating at the hip-joint.

*Resections.*

| | Recover'd. | Died. | Doubtful. | Total. |
|---|---|---|---|---|
| Shoulder-joint, | 5 | 1 | | 6 |
| Elbow-joint, | 3 | 1 | | 4 |
| Parts of hand, | 1 | | 1 | 2 |
| do. shaft of femur, | | 2 | | 2 |
| Knee-joint, | 1 | | | 1 |
| Parts of fiblua, | 1 | | | 1 |
| do. foot, | 1 | | | 1 |
| Total, | 12 | 4 | 1 | 17 |

*Ligations of Arteries.*

(Generally for secondary hæmorrhage.)

| | Recover'd. | Died. | Total. |
|---|---|---|---|
| Sub-clavian artery, | 1 | | 1 |
| Sub-scapular do. | 1 | | 1 |
| Facial do. | 1 | | 1 |
| Axillary do. | | 1 | 1 |
| Profunda femoris artery, | 1 | | 1 |
| Femoral artery, | 2 | 1 | 3 |
| Total, | 6 | 2 | 8 |

In reviewing these tables, it is a matter of profound regret that among some thousands of wounded, who, in different battles have been under the care of ourselves and others, we were able to trace out the results of so few cases; still, the careful observation of the facts here recorded, combined with statistics from other sources, will help to set at rest the most prominent of the disputed questions of military surgery.

The practical questions before the military operator are, mainly, the following:—

1. What cases require amputation?
2. What cases require resection?
3. What cases should be treated without operative interference?
4. What variations from accepted rules must be made, in view of special military exigencies.

First then:—

*What cases require amputation?*—The rule is now well established, that the military surgeon may go almost all lengths in his efforts to preserve superior extremities; but that in the inferior, amputation must be very extensively practiced.

*Amputation of the Shoulder-joint.*—This is only required in cases where an arm has been torn off by a cannon-shot, or otherwise so hopelessly disorganized as to render mortification of the whole limb inevitable. If the head of the humerus only is shattered, resection should be preferred. In our experience, as shown in the above tables, amputations at the shoulder have had a mortality of one in three, while resections of the joint only showed a loss of one in six.

In the Schleswick, Holstein, campaign, ESMARCH gives the results of 19 resections of the shoulder, of which, 12 recovered, and 7 died. GUTHRIE quotes 44 cases of amputation at the shoulder-joint, in the British Wars with Napoleon, of which, 17 died. Combining all these statistics, we find the following results:—

| | Total. number. | Recover'd. | Died. | Per cent of deaths. |
|---|---|---|---|---|
| Amputations at shoulder, | 50 | 31 | 19 | 38 |
| Resections of do. | 25 | 17 | 8 | 32 |

Showing an advantage of 6 per cent in favor of resections.

In addition to the diminished risk, the great value of the preserved limb is to be taken into account. After resection, the use of the elbow and hand is perfect; and some soldiers have even returned to duty as soon as the cure was perfected. In case of doubt whether an arm can be saved, time should be taken to watch the progress of the patient before deciding, for, although primary operations are preferable, yet the secondary ones are very well borne; and it is a man's duty to risk his life to some degree, for so important a member as a superior extremity. GUTHRIE fully sanctions the same opinion, when he affirms that amputations of the superior extremity should not be primary, unless the impossibility of saving the limb is obvious.

Sabre cuts and bullet wounds, simply opening the shoulder-joint, without serious comminution of the bone, do not render either resection or amputation necessary, as the patient recovers with anchylosis, in the majority of instances. If, however, the head of the humerus is badly comminuted, an operation of some kind is absolutely required, as the mortality in cases treated simply by splints, is found to be over 60 per cent.

*Amputations of the Arm.*—These should only be performed when there is no possibility of preserving the limb. Amputations for bad fractures of the humerus, or for shattered elbows, while there is still a good pulse at the wrist, are no longer justified by any respectable authority. It is often astonishing to inexperienced surgeons to see from what terrific injuries a wounded arm will recover itself. If the bone is shattered, the artery cut, and the anastomotic vessels also so extensively destroyed, that circulation in the limb ceases, amputation should be immediately resorted to. If, however, circulation continues in some measure below the injury, the loose fragments of bone should be picked out, and the limb dressed as for other compound fractures.

The mortality after amputations of the arm is but slight; of 11 cases in our records, not one died. Of 72 cases mentioned by GUTHRIE, only 17 died. Combining these statistics, we have the following result:—

| | Total number. | Recover'd. | Died. | Per cent of deaths. |
|---|---|---|---|---|
| Amputations of the arm, | 83 | 66 | 17 | 20½ |

*Amputations in the Fore-arm and Hand.*—As we recede from the body, both operations and injuries become less fatal. All the cases of amputation of the fore-arm and hand, of which we could obtain the results, recovered. The few who die, succumb not to the operation, but to the secondary effects of the deadly air of overcrowded hospitals. In every case where required, the amputation may be resorted to without fear; but it should be borne in mind that the fore-arm and hand recover from the most frightful looking wounds with surprising ease, and that every inch which can be preserved is of priceless value to the patient. In a mangled hand, almost every part which is not torn off may be preserved, and should be, generally, retained. We make these remarks, because we have observed that inexperienced surgeons will often be moved by the ghastly appearance of a fractured and lacerated hand, to undertake very unjustifiable amputations.

*Amputations at the Hip-joint.*—No case of this fell under our notice, as we all adopted the principle, that it was an operation which can scarcely ever be justified.

*Amputations of the Thigh.*—In this part of the body, we reverse the rules applied to the superior extremity. Instead of going all lengths to save the member, we incline more decidedly to prompt and resolute amputation on the field. Secondary amputations of the thigh are usually fatal, therefore, the decision of the surgeon must be made up on the spot, from the appearance of the case, and resolutely carried out. Our records show 20 amputations of the thigh, of which, 9 died, 10 recovered, and 1 remained doubtful, being a mortality of about 45 per cent. It is of the utmost importance here to observe the difference of mortality between the upper and lower parts of the thigh, because, on this difference are based life and death decisions. The following table illustrates it:—

| | Total cases. | Recover'd. | Died. | Doubtful. | Per cent of deaths. |
|---|---|---|---|---|---|
| Amputated upper 3d of thigh, | 5 | 1 | 4 | | 80 |
| do. middle do. | 4 | 2 | 2 | | 50 |
| do. lower do. | 11 | 7 | 3 | 1 | 27 |

Showing plainly that "every inch by which this operation approaches the body, increases its danger."

According to LONGMORE'S statistics, a similar percentage was observable in the Crimean Campaign, as is shown by the following table:—

| | Per cent of deaths. |
|---|---|
| Amputation, upper third, in Crimean War, . . . . . | 87 |
| do. middle do. do. . . . . . | 60 |
| do. lower, do. do. . . . . . | 57 |

These figures show a more favorable result in our army than in the British, by an average of about 20 per cent. Combining the two tables, we have approximately the following:—

| | Per cent of deaths. |
|---|---|
| Mortality of amputation at upper third, . . . . . . | 83½ |
| do. do. middle do. . . . . . . | 55 |
| do. do. lower do. . . . . . . | 42 |

The obvious deduction of which is, that the amputation should be made as far from the body as the nature of the injury will possibly permit. Such being the frightful mortality of amputations of the thigh, we tried in two cases to produce a better result, by resecting the ragged ends of the broken femur, and then treating it as for compound fracture. Both these cases died within the fifth day. The same experiment was tried on the Potomac, by Eastern surgeons, and also in the Crimea, and always with the same result,—every case proving fatal.

Still, other experiments have been made, by treating the case simply as a fracture, without any other operation than an incision to evacuate the pus. STROMEYER quotes 4 cases of recovery. Our tables show 9 cases treated in this manner, of which, 3 recovered, and 6 died. These cases were mostly fractures above the middle; hence the mortality of 66 per cent is not greater than would have followed amputation in the same place. In Europe, after the battle of Toulouse, this mode was tried on 43 of the most favorable cases, with a mortality of about 60 per cent, which, on the whole, is not much worse than the results of amputation, which, in nearly all fractures of the femur, must be as high as the middle, and has a mortality of 55 per cent.

A careful and very deliberate examination of this whole matter, has settled in our mind the following conclusions:—

1st.—A very large portion of the cases with badly comminuted femurs, will die within five days,—under all treatments, alike. There is no perfect reaction.

2d.—Shots through the spongy tissue of the trochanter and neck of the femur, are less fatal than those through the compact tissue of the shaft. This is contrary to STROMEYER'S opinion; but it is nevertheless true. The splintering of the bone, and consequent injury of soft parts, is far less in this spongy part than in the ivory-like shaft below. These cases of fractured neck require neither amputation nor resection of the head of the femur, a large part of them will recover with simple extension-splints and, in some cases, incisions to evacuate pus; whereas, amputations and military excisions at the hip-joint may be practically said to be all fatal. We know of 2 cases of this fracture which recovered without difficulty in straight splints.

3d.—Amputations above the middle of the femur should only be resorted to in desperate circumstances, where the limb below is either torn off or is so injured that it has but little prospect of escaping mortification. If the circulation and innervation are good below, a free incision should be made down to the comminuted bone, and the limb be dressed with a straight splint and adhesive-strap extension-bands. The case is a desperate one, but we are confident that this treatment will save more lives than amputation above the middle.

4th.—If amputation can be made below the middle of the thigh, it should be promptly performed, for all severe compound fractures of the lower half of the shaft of the femur, and all gun-shot fractures of the knee-joint. By this treatment, about 75 per cent of the patients may be saved; but if attempts are made to save the limb, almost every man will die. At the battle of Shiloh, a large number of cases were treated with this false conservatism, and many lives sacrificed in consequence. If any young surgeon feels reluctant to sacrifice a fair and plump thigh, for a mere little bullet hole of very harmless appearance in the knee, we advise him first to amputate, and afterwards to dissect the limb; he will find within the joint a horrible disorganization, such as no man can reasonably hope to survive, without operative assistance.

*Amputations of the Leg.*—These may be resorted to whenever a useful limb cannot be preserved, as the operation is not excessively dangerous. If, however, the circulation in the foot continues, and a chance of future usefulness of the member presents itself, conservative surgery should be practiced; because the danger of postponing or omitting amputation is not great, even though the foot should mortify. One hint may serve to guard young surgeons against a natural error: when a bullet traverses through the tibia from before, backwards, the front opening in the skin is small, but the fragments of the bone are driven back among the tissues of the calf, producing more danger of mortification than the first glance indicates. On the other hand, if the ball has traversed from behind, forwards, it drives all the splinters outward through the skin in front, doing less real injury than in the former case, but still tearing open the skin, and everting the flesh over an area of two or three inches in diameter. The wound looks so hideous, that it is not uncommon for the inexperienced operator to be moved by it to cut off the better limb and save the worse.

*Amputations of the Foot.*—These may be decided upon and executed by the same rules as in civil surgery.

*Resection of the Shoulder-joint.*—The grounds of choice between this and amputation have already been discussed under the head of "Amputations at the Shoulder." It is to be preferred, in proper cases, both for its superior safety, and because it saves a most important limb.

*Resection of the Elbow.*—Our lists show 4 cases of this resection, of which, 3 recovered, and 1 died. ESMARCH quotes 40

cases, of which, 6 died. Combining the two sets, we have this table:—

| | Number of cases. | Recover'd. | Died. | Per cent of deaths. |
|---|---|---|---|---|
| Resections of elbow-joint, | 44 | 37 | 7 | 16 |
| Amputation of arm, | 83 | 66 | 17 | 20½ |

Showing an apparent advantage of 4½ per cent in favor of resection. As amputation, however, was often for severer injuries than those which required resection, it will, probably, be fair to assume that in injuries which admit of the choice, the risks of the two operations are about equal; but as resection preserves, and amputation loses the hand, the choice is unquestionably for the former. We, therefore, advise resection for all comminuted gun-shot fractures of the elbow-joint, in which the preservation of the hand is not hopeless from gangrene.

*Resections of parts of the Hand.*—These should be governed by the same rules as in civil practice.

*Resections of the Knee-joint.*—The great mortality of amputations of the thigh, has caused this operation to be proposed as a substitute in cases of bullet wounds of the knee. Our tables show only one case, and that recovered. From all sources, European and American, we are able to collect accounts of only 8 cases in military practice, of which, 2 recovered, and 6 died; a mortality of 66 per cent, which is 24 per cent worse than that of amputations at the lower third of the thigh. More extensive statistics, however, are needed to settle its true value. At present, we advise, both from our own observations and careful review of the opinions of other surgeons, that in case good air and freedom from motion can be had for the patient, resection of the knee may be preferred; but, if he must be transported far in an ambulance, or put in a crowded hospital, where there is less than 1200 cubit feet of fresh air for each patient, resection will prove fatal. Amputation should then be at once performed, for delay with a view to secondary resection is not to be thought of.

*Resections in the Leg and Foot.*—These are well borne, and follow the same rules as in civil practice.

*Diseases of overcrowded Hospitals.*—There is a class of deadly complications following the injuries of patients after nearly every large battle, which are almost solely the product of overcrowding and bad air. These are the following:—

| | |
|---|---|
| Erysipelas, | Pyæmia, |
| Diffusive phlebitis, | Hospital gangrene. |

About 10 or 15 per cent of the deaths in military surgery are from these causes, and I regret to say, that in many instances these dead are slain by the surgeon, whose stupid ingenuity was all expended in procuring beds in warm and close quarters, where the patients poison each others' blood, instead of having free air where they may breathe and live.

We have observed with pain, that, partly by military necessity, and partly by ignorance of ventilation displayed by surgeons, this error of overcrowding is repeated after almost every large battle, and perpetuated in most of our large General Hospitals.

If the weather is not so inclement as to endanger death from cold, we have no doubt that by far the best plan is to keep the patients dispersed for two or three weeks in open tents and booths in the field; for, although in this way they have less comfortable beds, and coarser food than in Post Hospitals, they get fresh air, and with that they often survive the most desperate wounds.

It is often remarked, that men wounded in occasional skirmishes, where they are kept with the Regimental Hospital in the field, seldom have erysipelas or pyæmia, and recover from their injuries far more readily than those sent away to large, square, six story buildings, like the Overton Hospital in Memphis, where overcrowding is frequently unavoidable, and perfect ventilation an impossibility.

The results of our observations in the army, under this head, may be summed up, therefore, in one sentence:—Let the military surgeon see that he gets fresh air for his men in preference to food, warmth, or shelter.

Men will lie in snow, on wet ground, or under open sheds, and do well on bacon and hard bread; but, in close hospitals they will die, though they have all the luxuries of the world around them.

The principal military hospitals, within the bounds of our State, have been located at Mound City, Cairo, Quincy, Alton, Camp Butler, and Camp Douglas. The hospitals at Mound City and Quincy mostly received sick and wounded from the army in the field, while the others were simply the receptacles of the sick of the local camps. The Rebel sick were mostly at Camp Butler and Camp Douglas, and for some reason seemed to be entirely inferior to Northern men, in recuperative power. At Camp Douglas the ratio of mortality among them was double that of our own soldiers, although they had the utmost care and attention. It would be an important point added to our knowledge, to ascertain whether this difference arose from constitutional difference in vigor, previous bad hygienic management, or mental depression incident to being captured.

The writer of these pages only learned, after his arrival at Jacksonville, that no Report had been forwarded by the Committee on Surgery. He was, therefore, obliged to prepare this in the utmost haste, and without any opportunity for consultation with the other members.

